# The perceptions of hematopoietic stem cell transplantation decision-making in patients with hematologic malignancies: a qualitative systematic review

**DOI:** 10.1007/s00520-026-11381-8

**Published:** 2026-10-23

**Authors:** QingYing Wu, DanQi Chen, Fei Yuan, ShuQi Huang, Pan Fu, Ying Zhang, HuaFen Wang

**Affiliations:** 1 Department of Nursing, The First Affiliated Hospital, Zhejiang University School Of Medicine, 1367 Wenyi West Rd, Yuhang District, Hangzhou, 311121 Zhejiang Province China; 2 Department of Hematology, The First Affiliated Hospital, Zhejiang University School Of Medicine, Hangzhou, Zhejiang China

**Keywords:** Hematopoietic stem cell transplantation, Decision-making, Qualitative research, Systematic review

## Abstract

**Purpose:**

Hematopoietic stem cell transplantation (HSCT) remains the primary curative option for many hematologic malignancies, yet its substantial risks make the treatment decision highly complex and high stakes for patients. Although current qualitative studies have explored how patients perceive HSCT, a comprehensive synthesis of these insights is still lacking. This study synthesizes existing research on patient perceptions of HSCT to guide shared decision-making in clinical practice.

**Methods:**

A literature search was conducted across eight databases: PubMed, CINAHL, PsycARTICLES, Web of Science, Embase, the Cochrane Library, Scopus, and ProQuest Dissertations & Theses Global, spanning from their respective inceptions to February 2026. The Joanna Briggs Institute criteria for qualitative research were utilized to evaluate the quality of the studies, and a content analysis approach was employed to integrate the findings.

**Results:**

A total of 20 studies were included, yielding 4 new integrated findings: (1) constrained choices; (2) decision-making embedded in relational contexts; (3) the hidden toll: financial constraints and logistical hurdles; (4) concerns about restricted social participation and threats to identity caused by transplantation.

**Conclusion:**

HSCT decision-making is shaped not only by clinical risks and benefits, but also by constrained choice, relational contexts, practical circumstances, and concerns about future life. SDM-based decision support should therefore extend beyond information provision to clarify patients’ understanding of the decision space, identify factors that constrain choice, and address the values and longer-term concerns that matter to them.

**Supplementary Information:**

The online version contains supplementary material available at https://doi.org/10.1007/s00520-026-11381-8.

## Introduction

Over the past three decades, the number of hematologic malignancies has increased steadily worldwide, resulting in a major public health burden [1]. Projections indicate that global cases will reach 4.63 million by 2030 [2]. Unlike solid tumors, these diseases progress unpredictably [3]. Hematopoietic stem cell transplantation (HSCT) remains the only curative option for many hematologic malignancies [4]. The procedure transplants autologous or allogeneic stem cells to rebuild the recipient’s hematopoietic and immune functions. Its utilization has steadily increased over time [5]. Contemporary CIBMTR data similarly demonstrate improving survival across a diagnostically heterogeneous HSCT population. Three-year overall survival after allogeneic HSCT increased from 55.8% for procedures performed in 2012 ~ 2016 to 62.1% in 2017 ~ 2022, while survival following autologous HSCT increased from 79.6 to 82.6% [5]. Parallel to these gains in survival is the persistence of transplant-related risks [6]. A CIBMTR analysis of 4429 adult patients who received a first HSCT for hematological malignancies found that, although severe chronic graft-versus-host disease was associated with the lowest risk of relapse, it was simultaneously accompanied by a higher risk of non-relapse mortality, ultimately manifesting as shorter overall survival [7]. Infection represents another major fatal complication after transplantation. A large registry analysis by the EBMT of 232,618 patients who underwent HSCT showed that infection was the leading cause of death within 100 days after transplantation; although infection-related mortality declined over time, the risk of late infection-related death persisted [8]. Beyond mortality risks, transplant survivors consistently report profound physical, psychological, and social challenges [6, 9]. They endure prolonged recovery periods, face restrictions in daily activities, and frequently encounter social stigma and discrimination [10].Given the profound impact of HSCT on survival, patients must carefully deliberate when deciding whether to undergo the procedure [11]. HSCT therefore represents a complex and preference-sensitive decision, in which potential survival benefits must be considered alongside treatment-related risks, uncertainty, and their implications for patients’ lives.

Charles et al. defined Shared decision-making (SDM) as a deliberative, bidirectional process in which clinicians and patients jointly participate, exchange information, and reach a mutually agreed decision [12]. SDM is particularly suited to preference-sensitive decisions, because the appropriate choice depends, in part, on patients’ own values and preferences. The Ottawa decision support framework (ODSF) further operationalizes SDM by specifying the decision needs that decision support should address, including knowledge, outcome expectations, valued outcomes, decision-making role, and available support and resources [13]. By providing decision support tailored to these needs, an optimal decision should be both well-informed and consistent with the treatment outcomes and personal values that matter most to the patients [13]. How patients understand and appraise HSCT through the lens of SDM and decision support is therefore not merely supplementary information within the decision-making process, but a critical basis for judging whether their treatment choices genuinely reflect their personal values, goals, and priorities [14–16]. For example, patients may focus heavily on the hope for a cure and avoid fully considering potential complications and consequently experience severe psychological distress when unexpected side effects occur. This gap between expectation and reality can undermine patients' ability to adjust when unexpected clinical events arise [17, 18]. Furthermore, during the decision period, patients often worry about the treatment process, financial burdens, and their changing social roles. These concerns can directly trigger decisional conflict and subsequent regret [19], as well as compromise long-term treatment adherence [6].

The latest guideline from the European Society for Blood and Marrow Transplantation highlights the importance of understanding the patient’s perspective during decision-making. The guideline recommended initiating transplant consultations at the time of diagnosis [20]. Similarly, Cusatis [21] pointed out that merely focusing on psychological experiences after transplantation is insufficient, understanding their perspectives during the decision-making process is equally vital. However, few studies have thoroughly explored experiences during the decision-making phase [22]. Crawford [23] synthesized the literature and social media data to describe how patients view intensive treatments (such as transplants and high-dose chemotherapy) versus non-intensive options (such as palliative care and low-intensity chemotherapy). However, that study discussed HSCT merely as one of several intensive options, it failed to examine the unique decision-making context of HSCT independently.

In recent years, an increasing number of studies have focused on how patients with hematologic malignancies view the HSCT decision. These studies explore how patients understand the transplant, why they accept or decline it, their real experiences during the decision-making process, and how they balance hope and fear when considering this treatment [19, 24, 25]. To our knowledge, no review has yet synthesized these existing qualitative studies. Because patient perspectives substantially impact decision quality and subsequent outcomes, the lack of a systematic synthesis limits clinical understanding. This gap may hinder the development of comprehensive interventions to support patients during decision-making. Therefore, this qualitative systematic review aims to synthesize and reveal the perspectives of patients with hematologic malignancies during the HSCT decision-making process. The findings will help healthcare professionals provide targeted decision aids. Ultimately, this support can enhance patients’ satisfaction, reduce decisional conflict, and ensure that treatment choices align with personal values.

## Methods

### Study design

This review followed the Joanna Briggs Institute (JBI) methodology for systematic reviews of qualitative evidence [26]. Furthermore, the findings were reported according to the Enhancing Transparency in Reporting the Synthesis of Qualitative Research (ENTREQ) guidelines [27]. The study protocol was prospectively registered in PROSPERO (CRD420261334153).

### Search strategy

A literature search was conducted across eight electronic databases: PubMed, CINAHL, PsycARTICLES, Web of Science, Embase, the Cochrane Library, Scopus, and ProQuest Dissertations & Theses Global. To comprehensively capture how patients with hematologic malignancies perceive and experience the HSCT decision-making process, no lower limit was placed on publication year, and the search spanned from each database’s inception to February 2026. This decision was made deliberately, as HSCT decision-making is a relatively narrow field of qualitative inquiry in which relevant patient perspectives may be dispersed across earlier foundational studies and more recent work; restricting the search by date risked excluding conceptually important accounts. The search strategy employed a combination of Medical Subject Headings (MeSH) and free-text keywords, integrated using Boolean operators. To ensure search comprehensiveness, the reference lists of all included studies were manually screened for additional relevant publications. The detailed search strategy is provided in Supplementary Material 1.

### Inclusion and exclusion criteria

The PICoS framework was used to establish the study selection criteria. Inclusion criteria are as follows: (1) population: adult patients (aged ≥ 18 years) diagnosed with hematological malignancies. For multi-stakeholder studies (e.g., involving physicians, nurses, or caregivers), patients had to comprise at least 40% of the interviewees. (2) Phenomenon of interest: patients’ subjective perspectives during the HSCT decision-making process, specifically capturing the influencing factors and their psychological experiences. (3) Context: the HSCT decision-making environment. This encompassed patients currently navigating the choice, alongside those reflecting on their past decisions—regardless of whether they ultimately accepted or declined the transplant. (4) Study design: research explicitly employed qualitative methods or mixed-methods designs with a distinct qualitative component (e.g., descriptive qualitative research, phenomenology, grounded theory). Exclusion criteria are as follows: (1) duplicates, conference proceedings, reviews, correspondence, abstracts, and non-English publications. (2) Irrelevant articles, defined as studies that did not explore patients’ perspectives on, experiences of, or factors influencing the HSCT decision-making process (e.g., studies reporting only transplant outcomes, survival, complications, or post-transplant quality of life without addressing the decision-making experience). (3) Studies failing to report patients’ HSCT decision-making experiences separately from those of other stakeholders.

### Quality appraisal

Prior to data extraction and synthesis, we specified that the quality appraisal would be used to identify methodological strengths and limitations of the included studies and to inform interpretation of the synthesized findings, rather than to determine eligibility. Accordingly, no numerical cut-off score was prespecified for study exclusion on the basis of methodological quality; instead, appraisal results were used to inform confidence in the synthesized findings. Consistent with the position that methodological quality should inform rather than determine inclusion [28], no study was excluded solely because of poor methodological quality, provided that it contributed data relevant to the research aim. To determine the quality of the included studies and to enhance the reliability of the synthesis, two researchers (WQY, HSQ) independently appraised the selected studies using the JBI critical appraisal checklist for qualitative research [26]. This tool comprises ten items assessing the congruence between methodology and philosophical underpinnings, the alignment of research objectives with methods, the selection of data collection and analysis approaches, the consistency of result interpretation, researchers’ reflexivity, the representation of participants, and adherence to research ethics. Each item was rated as “yes,” “no,” or “unclear.” Any disagreements were resolved through discussion and consensus within the research group.

### Data extraction

Data were extracted from the eligible studies using a pre-specified Microsoft Excel spreadsheet, including author, year of publication, country, participants, study design, phenomenon of interest, data collection and analysis methods, and key findings. This process was completed by the first author (WQY) and subsequently verified by a second researcher (CDQ) to ensure accuracy and completeness. Any discrepancies were resolved through discussion, and a third researcher (WHF) was consulted when consensus could not be reached.

### Data synthesis

Data synthesis was performed using the content analysis approach proposed by Elo and Kyngäs [29]. This method effectively processes large volumes of textual data and integrates multiple sources to corroborate evidence. Furthermore, given the scarce and fragmented prior knowledge regarding HSCT decision-making, an inductive approach was adopted for the synthesis [30], allowing categories to emerge from the data rather than imposing a predefined analytical framework. The material for analysis comprised findings reported in the results sections of the included studies that addressed patients’ views and experiences of HSCT decision-making and factors influencing their decisions, including participants’ original quotations and the primary study authors’ interpretations.

The first author (WQY) initially read the extracted findings repeatedly to become familiar with the data as a whole. Within the extracted material, meaning units—phrases, sentences, or paragraphs conveying a distinct meaning relevant to the review question—were identified and subjected to open coding. Codes were generated inductively and kept close to the meaning of the original data. Codes were iteratively compared within and across studies to identify similarities, differences, and recurring patterns. Codes with similar meanings were grouped into subcategories, while divergent or opposing perspectives were retained to reflect variation across studies. Subcategories were subsequently compared according to their shared characteristics and conceptual relationships and grouped into higher-order categories. Through progressive abstraction, categories were labelled to capture their underlying meanings, resulting in a final category structure addressing the review question.

Initial coding, grouping, and category development were undertaken by WQY. CDQ subsequently reviewed the analytical process by systematically examining the correspondence between the original data, codes, subcategories, and final categories. Disagreements concerning code labels, grouping, or category boundaries were resolved through discussion; where consensus could not be reached, WHF was consulted. Microsoft Excel was used to organize the extracted data, meaning units, codes, subcategories, and categories and to support iterative comparisons within and across studies. No dedicated qualitative data analysis software was used.

### Assessing confidence in the findings

Confidence in each synthesized finding was assessed using the GRADE-CERQual approach. One reviewer (WQY) conducted the initial assessment, and a second reviewer (HSQ) independently checked the judgments and their supporting rationales. Each synthesized finding was evaluated across four domains: methodological limitations, relevance, coherence, and adequacy of data [31]. For each domain, the level of concern was judged according to the extent to which limitations might reduce confidence in the finding. The domain-level judgments were then considered together to determine an overall confidence rating of high, moderate, low, or very low. Any disagreements were resolved through discussion, with a third reviewer (WHF) consulted if consensus could not be reached. Confidence ratings were not used to exclude synthesized findings; rather, they were used to indicate the degree of certainty in each finding and to guide the interpretation of the review results. Detailed judgments and rationales are provided in Supplementary Material 2.

## Results

### Literature search

**Fig. 1:**
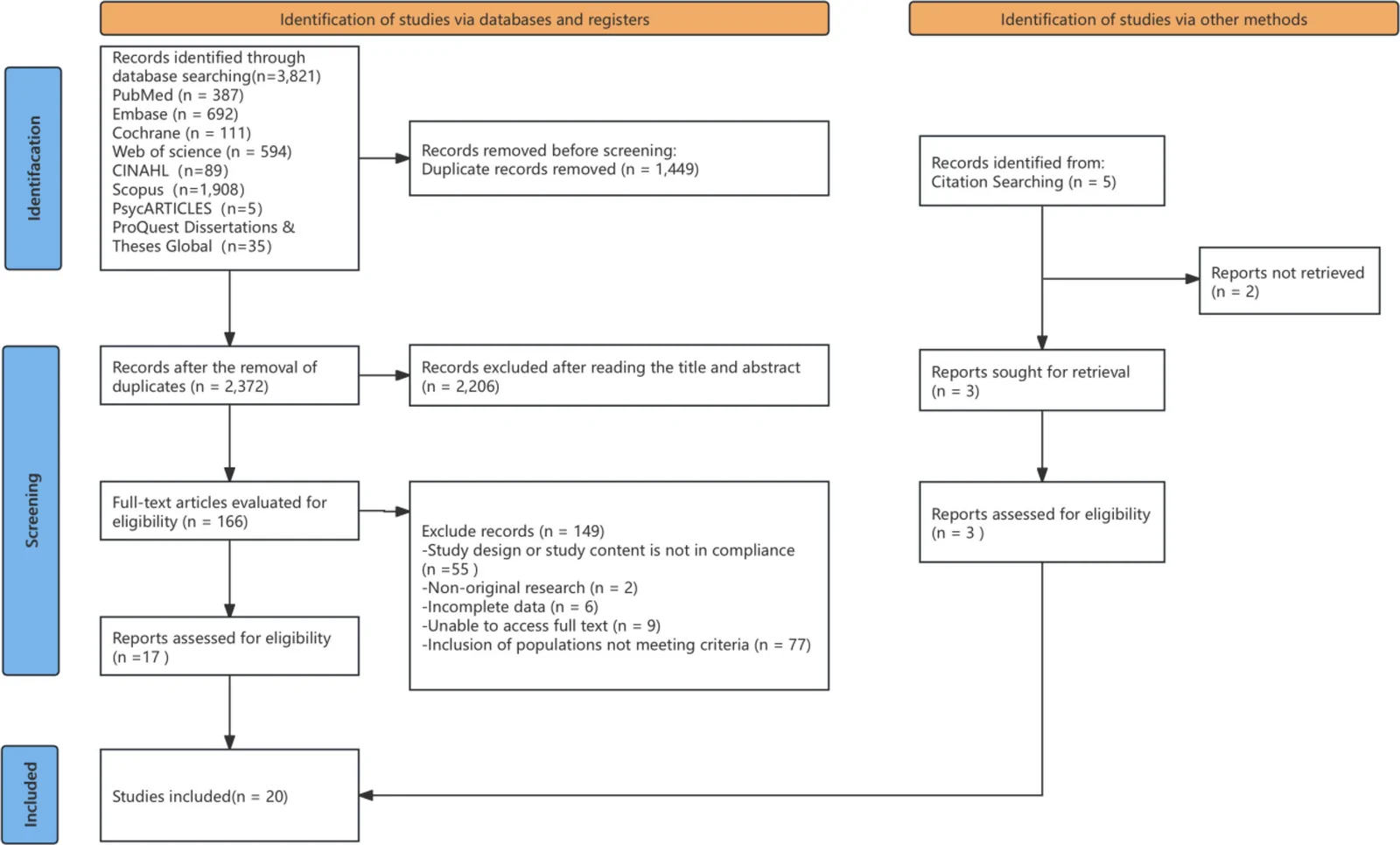
PRISMA flow diagram

Initially, 3821 articles were retrieved, and 2372 remained after removing 1449 duplicates. Upon reviewing the titles and abstracts, 166 articles were selected as relevant, and the other 2206 were discarded. The full texts of these articles were then reassessed, resulting in 17 articles being selected. A manual search of the reference lists identified three additional articles, yielding a final total of 20 articles being selected for quality evaluation. The PRISMA diagram is shown in Fig. 1.

### Quality appraisal

All the studies met at least 7 standard points, with the highest number of studies meeting 10, consequently(see supplementary material 3). Nine studies [17, 19, 32–38] demonstrated congruence between their philosophical perspectives and methodological approaches. Additionally, 4 studies [19, 32, 36, 38] explicated the researchers’ cultural backgrounds and addressed the potential influence on the research process.

### Characteristics of included studies

These 20 publications comprised 17 peer-reviewed articles (16 qualitative studies and one mixed-methods study) and 3 doctoral dissertations. The studies were conducted in the USA (*n* = 10), China (*n* = 2), Germany (*n* = 2), France (*n* = 1), India (*n* = 1), Vietnam (*n* = 1), the UK (*n* = 1), Australia (*n* = 1), and Canada (*n* = 1). A total of 1008 patients with hematological malignancies were included in the 20 studies. Among them, 336 patients agreed to undergo HSCT, 125 declined transplantation, and 547 were still considering HSCT or were undergoing the treatment decision-making process. Among the patients for whom the transplant type and decision status were explicitly reported, 62 patients agreed to undergo allogeneic HSCT and 20 agreed to undergo autologous HSCT. Twelve patients declined transplantation, including 1 patient who declined allogeneic HSCT and 11 who declined autologous HSCT. These studies applied explicit data analysis methods, including thematic analysis, content analysis, and grounded theory (see Table 1).

**Table 1:** Characteristic of the included studies

Author(s), year, country	Participants	Study design	Phenomenon of interest	Methodology: data collection and analysis	Main findings
Foster et al. [39], 2020, USA	35 patients received an aHSCT at least 3 months prior to study enrolment; the report did not distinguish allogeneic from autologous transplantation	Qualitative descriptive	To identify patients’ HCT related information needs to inform treatment decision making	Semi-structured focus group discussions, analyzed using thematic data analysis	(1) The need for tailored resources with age-specific recovery expectations
					(2) The need for the right amount of information at the right times
					(3) The benefit of peer support
Nowak et al. [24], 2025, Germany	8 patients undergoing the informed-consent process for alloHSCT, and 7 were interviewed after completing alloHSCT	Grounded theory	To explore the informed consent and decision-making process in the context of alloHSCT, with a particular focus on ethical challenges	Semi-structured, problem-centred interviews, analyzed using grounded theory	(1) No choice
					(2) The goal is the healing
					(3) Fear of dying and the desire to prolong life
					(4) Trust in medical expertise
					(5) Lack of knowledge about or not engaging with alternatives
					(6) It was clear from the start
Fiala et al. [32], 2019, USA	11 newly diagnosed myeloma patients declined the autoHSCT. Another 206 patients entered the quantitative cohort (of whom 129 received autoHSCT, 77 did not)	Mixed-methods study: qualitative descriptive	To focus on the decision-making with regard to alloHSCT and identify the reasons why patients with newly diagnosed myeloma referred to our center did not undergo autoHSCT	Semi-structured interviews, analyzed using analytic narrative	(1) Unclear gains of AHSCT versus standard chemotherapy
					(2) possible toxicity and disruption in quality of life;
					(3) Impersonalized medicine
Yuan et al. [19], 2024, China	16 patients’ eligibility for HSCT; the report did not distinguish allogeneic from autologous transplantation	Qualitative descriptive	To explore and understand the conflict in decision-making of hematopoietic stem cell transplantation in patients with hematological neoplasms	Semi-structured interview, analyzed using thematic analysis	(1) Weighing the pros and cons of HSCT
					(2) Financial burden versus desire for rebirth
					(3) Treatment urgency versus being unprepared
					(4) Saving oneself versus damaging loved ones and (5) Family companionship versus emotional isolation
Forsyth et al. [33], 2011, Australia	16 patients had agreed to undergo allo-BMT	Qualitative Descriptive	To fully appreciate the relational and embodied nature of decision-making	Interviewed, analyzed using broad coding framework	(1) Medical factors
					(2) Social factors
Sarradon-Ecket al. [36], 2024, France	22 alloHSCT recipients, 1 patient declined alloHSCT	Grounded theory	To identify the factors involved in overseas patients’ decision to accept alloHSCT	In-depth semi-directive interviews, analyzed using	(1)A constrained choice
					(2) Factors favoring patients' acceptance
					(3) Factors limiting patients' acceptance
Solanki et al. [40],2021, India	514 patients were considering of alloHSCT	Qualitative descriptive	To understand barriers, and support system while considering HSCT and the chances of getting matched unrelated donor for these patients	Semi-structured interview, analyzed using thematic analysis	(1) Cost of transplant
					(2) Availability of 10/10 HLA matched donor
					(3) Questionable quality of life after HSCT
					(4) No social acceptance after HSCT
					(5) Apprehension about side effect of HSCT therapy
					(6) Not sure of success
					(7) Fertility
					(8) Loss of job
					(9) Duration of stay in hospital
					(10) A friend
					(11) Coworker
					(12) Doctor or medical staff
					(13) The only option
					(14) Based on the belief in God
Liu et al. [25], 2025, China	13 patients had been offered auto-HSCT and made a treatment decision	Phenomenology	To comprehensively understand the experiences of shared decision-making regarding autoHSCT among older patients with hematological malignancies and physicians	Semi-structured, in-depth face-to-face individual interviews other qualitative approaches	(1) Perceived benefits and risks
					(2) Financial challenges, and family involvement
					(3) Mutual trust between patients and physicians
Bywater and Atkins [17], 2001, UK	7 BMT candidates interviewed within 1 month before their scheduled transplantation; the report did not distinguish allogeneic from autoHSCT	Grounded theory	To provide a more detailed theoretical analysis of factors influencing patients’ decisions to undergo bone marrow transplantation from a sibling or matched unrelated donor	Unstructured interviews, analyzed using grounded theory	(1) The logical mind
					(2) Gut instinct
					(3) Personal aims
					(4) Hope
					(5) Preparing for transplant
					(6) Trust in professionals
Hansen et al. [34], 2025, USA	14 patients underwent HSCT; transplant type was not specified in the participant description	Qualitative descriptive	Assessed how patients who have undergone HSCT and their caregivers make treatment decisions related to HSCT	Focus-group, analyzed using thematic analysis	Conversation; Consideration; Decision-making
					(2) Patients’ perceptions and knowledge-related themes: Emotion; Reflection; Information source
Mian et al. [41],2023, Canada	18 patients underwent autoHSCT	Qualitative descriptive	To explore factors that influence decision-making regarding HSCT as a treatment modality among older adults with multiple myeloma	Semi-structured telephone interviews, analyzed using thematic analysis	(1) Trust in oncologist
					(2) Autonomy
					(3) Family support
					(4) Additional information resources
					(5) Best treatment option
					(6) Afraid of side effects
Cytryn et al. [42], 2023 USA	29 patients (14 patients underwent autoHSCT)	Qualitative descriptive	To identify the reasons and needs underlying disparities in treatment among patients diagnosed with multiple myeloma	In-depth interviews, analyzed using inductive process	(1) Pro/con assessment
					(2) Physician recommendation
					(3) Patient individual preferences for decision making
Hansen et al. [43], 2023, USA	16 patients had underwent autoHSCT within the past 5 years	Qualitative descriptive	To explore multiple myeloma patients received an ASCT shared their experience	Focus group meetings, analyzed using thematic analysis	(1) Oncologists were the primary drivers in making
					(2) About half of the patients stated ASCT was their first recommended line of treatment
Wu et al. [18], 2026, USA	18 patients(8 received autoHSCT)	Qualitative descriptive	To access to ASCT in multiple myeloma, provides a much-needed multistakeholders perspective	Semi-structured interview, analyzed using content analysis	(1) Caregiver support
					(2) Patient concerns and knowledge
					(3) Financial toxicity
					(4) Logistics
					(5) Social support
Yanez et al. [37], 2023, USA	18 patients who had completed transplant in the past 12 months; transplant type was not specified in the participant description	Qualitative descriptive	To explore the lived experience of Hispanics/Latinx transplant recipients, before, during, and after transplant	Semi-structured interview, analyzed using content analysis	(1) Pre-transplant barriers and concerns
					(2) Complex relationships with medical teams
					(3) Informational mismatch
Reimer et al. [38] 2025, Germany	17 patients (4 patients were about to undergo alloHSCT; 7 patients were currently inpatient because of alloHSCT; 6 patients were in outpatient care for a maximum of 100 days after alloHSCT)	Qualitative descriptive	To explore the patients’ experiences considering their life threat in the context of the alloHSCT	Semi-structured interview, analyzed using content analysis	Facing the existential realities posed by diagnosis
					(2) The sole chance for survival
					(3) Uncertainty
Nguyen et al. [35], 2025, Vietnamese	22 patients (19 patients underwent HSCT)	Qualitative descriptive	Understand the decision-making experiences of adult patients with hematologic malignancies regarding HSCT through the perspective of relational autonomy	Online semi-structured interviews, analyzed using content analysis	(1) Coming to identify as a patient
					(2) Experiencing the influence of relational networks
					(3) Hoping one’s voice will be heard
Fadem [44], 2021, USA	7 patients underwent allo-BMT	Dissertations and thesis	Exploring and understanding the real experiences, confusions, and needs of patients during the BMT decision-making process	Semi-structured interview, analyzed using thematic analysis	(1) Unknowns (2) Timeline (3) Experiential information (4) Decision-making (5) Unknowns vs. Preparation
Kumasaka [45], 1991, USA	5 patients were considering undergoing BMT treatment; the report did not distinguish allogeneic from autologous transplantation	Dissertations and thesis	What factors influence the decision to undergo BMT	Semi-structured interview, analyzed using constant comparative analysis	(1) Motivation (2) Control (3) Information (4) Loss of control (5) Avoidance (6) Fear (7) Support
Haberman [46], 1987, USA	23 patients (19 patients underwent allo-BMT, 1 patients received auto-BMT)	Dissertations and thesis	Patients’ experiences and perspectives in deciding whether to undergo BMT	Semi-structured interview, grounded theory	Nothing else they can do Only hope for a cure Primary physician encouraged BMT Illness course is uncertain No other choice This is the best BMT center in world

*HSCT* hematopoietic stem cell transplantation, *autoHSCT* autologous hematopoietic stem cell transplant, *alloHSCT* allogeneic hematopoietic stem cell transplant

### Synthesis of findings

**Fig. 2:**
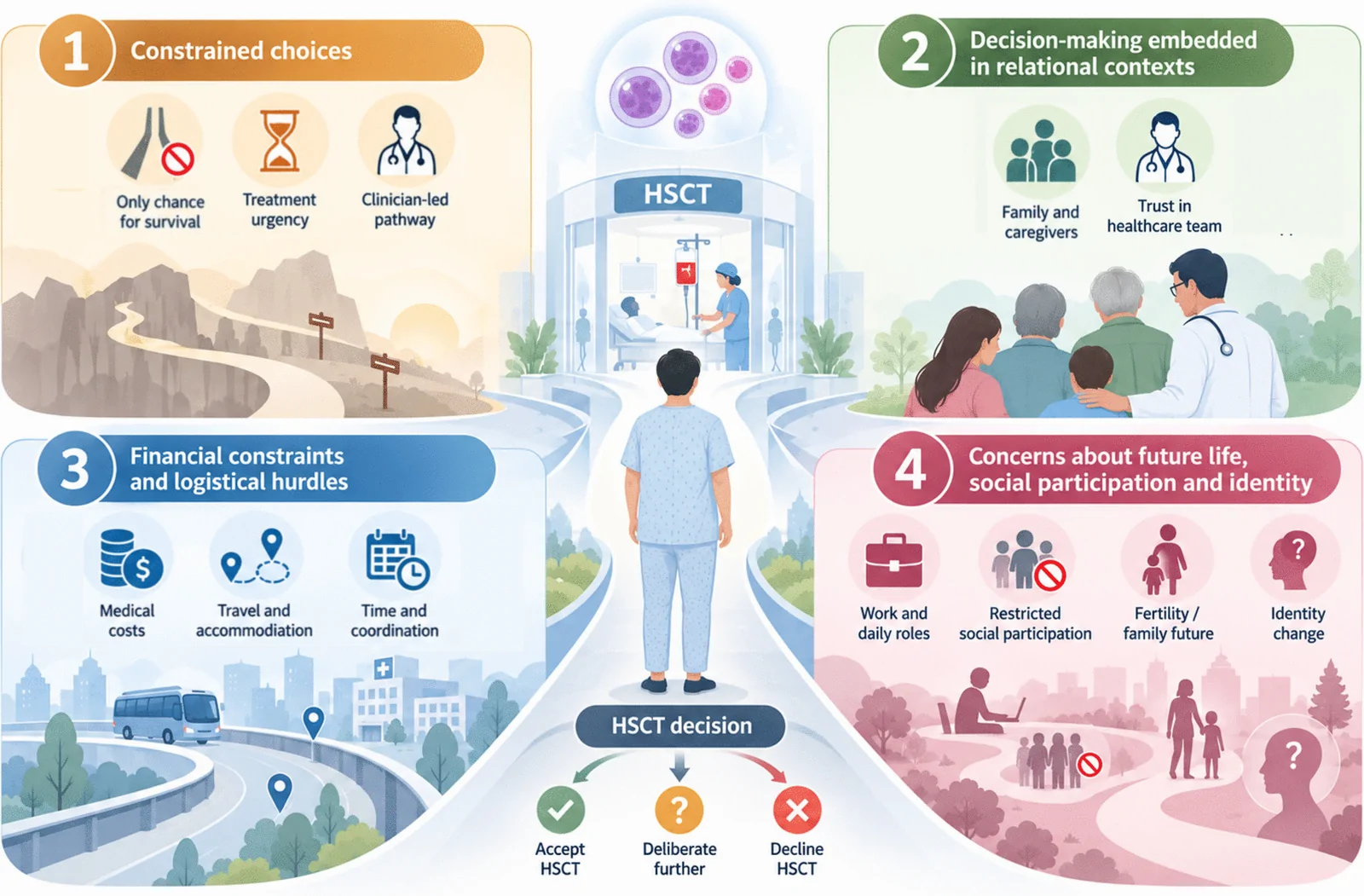
Synthesized findings on HSCT decision-making

The findings are organized into 4 core categories: (1) constrained choices; (2) decision-making embedded in relational contexts; (3) the hidden toll: financial constraints and logistical hurdles; (4) concerns about restricted social participation and threats to identity caused by transplantation. Each category is further specified through subcategories that illuminate patients’ perspectives on HSCT decision-making (see in Fig. 2).

#### Constrained choices

Patients with hematologic malignancies perceived that they did not decide on transplantation in a free and open context. Instead, they experienced this process as a restricted choice. This restriction manifested in two dimensions. Internally, it was constrained by the disease itself, the deadly nature of hematologic malignancies meant that HSCT was the only medical chance left for survival. Externally, it was constrained by the medical system: the authoritative attitude of doctors and the fast-paced treatment schedule bypassed the patients, making them feel that the decision had already been made for them without their active participation.

##### Constrained by the disease: the only chance for survival

Patients prioritized a cure above all other clinical goals [17, 24, 38, 44–46]. From their perspective, rejecting transplantation in favor of conventional chemotherapy equated to choosing a gradual path toward certain death. As one patient described, “Yes, but there is no other way for me to survive. Otherwise, I will definitely die. If you don't accept the transplant, you die, don’t you?” [44] Despite its high risks, HSCT offered the only possible pathway to a cure [17, 24, 33, 36, 38]. This reality ultimately drove patients to choose HSCT to regain hope [17, 24, 33, 36, 44, 46]. Because patients prioritized a cure and recognized HSCT as the sole means to achieve it, they viewed the procedure as their “only chance.” This perception profoundly generated a sense of having “no other choice.” [17, 24, 33] One patient vividly described this desperate hope: “It was a bit of a light at the end of the tunnel. With this aggressive form of cancer, I’m not going to live more than six or eight weeks, so it really was never an option. Because it is my only hope.” [33] Therefore, patients undergoing HSCT generally believed “I had no choice.” For them, transplantation never represented an option to weigh. Rather, it served as the only way out, which harbored their sole hope for survival [33].

##### Constrained by the medical system: no need to choose

This sub-theme captures how patients came to perceive that the decision regarding HSCT was not theirs to make, effectively removing their sense of choice. When facing the decision about HSCT, patients realized that they and their families lacked the knowledge to judge clinical options [33, 46]. Consequently, they often stated, “I can only hand myself over to the doctors and the treatment process.” However, during conversations with physicians, patients frequently heard: “You need a transplant, otherwise there is no other way.” [44, 45] Some patients also noted that the choice had already been made the moment doctors recommended the transplant [41]. Furthermore, tightly packed medical schedules created an illusion. Patients felt they no longer needed to decide whether to undergo HSCT at all, as illustrated by the following statement: “Then I no longer need to decide. The decision had already been made. So, that was too fast for my liking. There is no going back then, when suddenly the donor is already at the door. But well, now it has turned out that way.” [24] Given that hematologic malignancies progressed rapidly, patients faced severe time constraints to make a decision [19, 33, 40]. As one patient described: “He (the physician) said to me, ‘Of course you must do it, that’s how it is, and there’s no other way about it’. There was no shilly-shallying.” [36] 

#### Decision-making embedded in relational contexts

##### Beyond the patients: the family as the ultimate decision-making unit

Patients understood their decisions as closely tied to their roles and relationships within the family, rather than viewing themselves as independent decision-makers. Factors such as familial attitudes toward the procedure, responsibilities to the family, and the availability of caregivers profoundly shaped their choices [19, 33, 35]. First, encouragement and support from family members served as a crucial facilitator for accepting HSCT. Patients frequently cited their families’ reliance on them as a primary motivation to undergo the procedure [33]. As one patient articulated: “My children are very supportive of the transplant. Although my son is far away in Yunnan, he hopes I can live a little longer. He says that as long as the parents are at home, there is always a home to return to.” [25] At the same time, patients also considered how transplantation might affect the family as a whole. Some worried about becoming a burden to their children or disrupting family well-being, and these concerns could weigh against transplantation [19, 25, 37, 40, 41]. One patient who declined HSCT stated, “I already feel like a burden to my children. After chemotherapy, I just want a peaceful life and do not want to add to their burden.” [25] 

This dilemma was particularly evident among patients considering allogeneic HSCT. When family members served as potential donors, patients worried that the procedure might inflict physical harm on their relatives or even damage familial relationships [18]. One patient expressed this specific concern: “His wife thought that extracting the bone marrow would cause a lot of damage to his body. So, I am now torn and very scared that it will affect his family relationships.” [19] Alternatively, opting to wait for suitable stem cells from a bone marrow registry required significantly more time [19]. Confronted with this reality, the urgent need to survive and the deep-seated guilt toward family donors plunged patients into profound ambivalence during the process of decision-making.

Furthermore, the familial roles that patients occupied drove them to look beyond the medical intervention itself. Instead, they anchored their decisions about HSCT heavily on their responsibilities to the family [33, 36, 41]. A mother who accepted HSCT noted, “I am doing this mainly for them. My eldest daughter has been suffering from a chronic illness for the last four or five years, and she needs a mother to take care of her.” [33] Driven by the fact that their children were still young, many patients frankly admitted that they sought the transplant to continue fulfilling their parental duties [35–37].

To reduce the risks of infection and mortality, medical protocols required patients to undergo protective isolation, which prevented family members from consistently accompanying and encouraging them [19]. Consequently, some patients who declined a second transplant confessed that they refused to endure such loneliness and helplessness without their families’ presence again [19, 36]. Following discharge, rigorous hygiene management—such as strictly wearing masks and gloves, and remaining at home for extended periods—relied heavily on the involvement and collaboration of family members [39]. Therefore, the adequacy of the family support system emerged as a critical factor when patients evaluated the option of HSCT [40, 43]. Notably, anticipating a lack of familial caregiving post-transplantation prompted some individuals to refuse HSCT [18].

##### Trust in the healthcare team: a foundation for decision-making

Across the included studies, evidence consistently revealed that a robust foundation of trust between patients and the healthcare team emerged as pivotal to the decision-making process [24, 25, 34, 35, 40–43]. This trust was cultivated through multifaceted interactions and support provided by the medical team. Both physicians and nurses contributed to establishing this trust through their professional competence. Physicians’ expertise formed the bedrock of this trust. Primary studies indicated that patients perceived their physicians as authoritative figures capable of facilitating stem cell harvesting and formulating strategies to manage potential complications [43], Consequently, patients firmly believed that the healthcare team would invariably guide them toward the optimal clinical choice [24, 34, 41]. Therefore, physicians’ recommendations profoundly shaped patients’ perceptions and attitudes, whether they advocated for HSCT or alternative treatments such as chemotherapy [35]. Illustrating this reliance, one primary study quoted a patient stating: “I fully trust my doctors, my specialist…he has 30 years of experience, while I have no experience at all.” Conversely, when physicians recommended against HSCT based on pre-transplantation evaluations, patients readily accepted that this “non-recommendation” inherently disqualified the procedure as a viable path. This unwavering trust in medical authority spared patients from deciphering complex medical rationales, which ultimately enabled them to swiftly finalize their decision to forgo the treatment [42]. Patients also forged deeply respectful and trusting relationships with nurses. Participants consistently appreciated how nurses alleviated their anxiety throughout the decision-making trajectory [35, 45]. As vividly captured by a patient in a study: “The nurses at the transplant center are like the best machines in the world, they are there to help you, and you just have to trust them.” [39] 

#### The hidden toll: financial constraints and logistical hurdles

Facing both the direct exorbitant medical costs of the transplantation and the ensuing logistical expenses for commuting and accommodation, the patients experienced a compounding economic strain. This intersection of financial burdens significantly modulated the patients’ decision-making process and subjective experience of the transplantation [37, 40, 42]. These logistical hurdles are tightly intertwined with financial constraints to collectively shape their decision-making. Some patients noted that their ongoing treatments had already depleted their household savings; therefore, paying the “prohibitive” costs of HSCT would essentially plunge their families into a financial crisis [40]. This concern was particularly pronounced among older adults. Older patients who opted for chemotherapy frankly admitted that avoiding the exorbitant costs of HSCT directly prompted them to forgo the procedure [25]. Some patients reported that the availability of a family donor would make the decision to proceed with HSCT easier because they perceived it to involve less time and lower costs than transplantation from a matched unrelated donor [40].

For patients residing far from transplant centers, prolonged travel posed an immense strain, particularly as health insurance failed to cover hotel accommodations [37]. Not only did they have to coordinate transportation well in advance, but they also had to navigate the travel risks imposed by severe winter weather [18, 42]. A scarcity of transportation resources further exacerbated this predicament. Two patients without personal vehicles confessed that their access to shuttle services was strictly limited and required advanced booking, which rendered an already complex medical journey even more uncontrollable [42].

Consequently, these pragmatic logistical barriers occasionally deterred patients from choosing the transplant outright. As one patient articulated: “They knew it was going to cost a lot of money for me to go to Philly for the transplant, and it was a funding issue for me… because of the travel, the expense would be very high… That’s why I was more interested in doing the chemo here.” [42] Furthermore, patients requiring transnational transplants confronted even more severe geographical barriers. After relapsing in 2018, one patient hesitated to undergo an allogeneic transplant solely due to the extreme distance. She frankly admitted that if faced with the same situation again, she would never consent to the procedure [36].

#### Concerns about restricted social participation and threats to identity caused by transplantation

Concerns over post-transplant social implications profoundly shaped patients’ decisions regarding HSCT. Before committing to the procedure, they needed to understand how the transplant might alter their social identity [39]. Patients declining HSCT frequently voiced fears of facing social rejection post-transplantation [40]. Regarding fertility, the myeloablative conditioning regimen commonly used in HSCT can cause irreversible damage to the reproductive system [19]. As one young married female patient expressed, the drastically reduced chance of pregnancy following transplantation filled her with dread, and the dual social stigma of cancer and childlessness would deal a severe psychological blow [40]. Furthermore, unlike other antineoplastic therapies, patients following HSCT exhibit profound immune compromise and are at substantially elevated risk of infection. Consequently, medical recommendations typically necessitate strict limitations on outdoor activities, avoidance of crowded settings, and in many cases, prolonged cessation of work to minimize physical exertion and reduce infectious exposure [33], patients worried that the prolonged recovery period will permanently end their careers. This anticipated detachment from their established social roles severely restricts their future social participation [32]. Additionally, physical changes induced by the transplant threaten patients’ social identity. They deeply desired others to still view them as their former selves, simply as individuals who have conquered a disease. One patient who ultimately declined allo-HSCT described the graft as a threat to bodily identity, stating, “I don’t feel like putting things from other people into my body. I don’t want them to be mixed up with mine.” [36] Such bodily anxieties may exacerbate their overarching fear of failing to reintegrate into society under their original identity.

## Discussion

This systematic review involved the identification, review, and synthesis of 20 qualitative studies exploring the perceptions of patients with hematologic malignancies when deciding whether to undergo HSCT. Patients did not merely weigh competing medical options but had to navigate a constrained choice driven by the severe mortality threat of the disease and the urgency of the medical system. Their decision-making process was deeply embedded in relational contexts, relying heavily on the family. Furthermore, patients also weighed profound concerns about restricted social participation and threats to their social identity. At the same time, they were forced to confront the hidden toll of the treatment, including intersecting financial constraints and logistical hurdles.

This study identified that patients with hematological malignancies often experience a pronounced sense of “having no choice” when considering HSCT. Similar experiences of “having no real choice” have been reported among patients with diffuse cutaneous systemic sclerosis, in whom HSCT was regarded as the most promising, or even the only feasible treatment option when the disease deteriorated rapidly, and previous therapies failed to achieve disease control [47]. Taken together, these findings suggest that constrained choice in HSCT decision-making may exist across different disease contexts and should not be simplistically understood as a phenomenon unique to hematological malignancies.

However, the degree of perceived choice appears to differ across disease contexts. Compared with the findings of this study, patients with non-malignant conditions (e.g., those with sickle cell disease) more often described consideration of transplantation as an ongoing process of weighing: patients assessed when the risks of transplantation became acceptable in light of their current disease burden, progression of complications, and the effectiveness of alternative treatment options, and some took a considerable time to reach a final decision even after transplantation was first presented as an option [48]. We therefore speculate that HSCT decision-making can be understood as lying along a continuum ranging from deliberative, weighed choice to constrained choice. Patients’ position along this continuum may be shaped by the combined influence of factors such as the rate of disease progression, the availability of acceptable alternatives, the urgency of treatment, and the time available for deliberation. Within this framework, what may characterize patients with hematological malignancies is not a unique experience of “having no choice”; rather, as the tumor continues to progress and a perceived threat of imminent death, transplant assessment, donor preparation, and treatment scheduling move forward, the time available for patients to reconsider different options or change their decision may be further compressed. Under these circumstances, patients with hematological malignancies may be more readily pushed towards the constrained end of this continuum.

It is also important to note that this decision-making process may be accompanied by a significant optimistic bias [36]. Previous research showed that patients who undergo HSCT often hold higher expectations of treatment benefits, tending to underestimate transplant-related mortality while overestimating the chances of cure [3, 49], which may lead them to be overly optimistic about returning to a normal life after transplantation, while overlooking other important information [4, 50]. These findings highlight the need for targeted patients’ education to correct such cognitive biases and foster a more comprehensive understanding of HSCT.

Notably, the notion of a “constrained choice” frequently leads patients to relinquish decisional responsibility to medical authorities [51]. A considerable proportion of patients with hematological malignancies prefer to adopt a passive role in decision-making [33], by placing complete trust in medical advice, they gained a sense of security [41]. Patenaude [52] observed that when oncologists provided information about HSCT, they rarely discussed alternative treatments. Instead, patients heard directive statements such as “this is the standard approach” or “this is what we will do.” Such directive communication may create a sense that the decision has already been made by clinicians, leaving patients with a primarily compliant role [44].

This raises a further concern. The fact that patients ultimately provide informed consent does not, in itself, demonstrate that their preferences and values have been adequately integrated, nor does it establish that shared decision-making has been achieved [12]. From an SDM perspective, the key issue is whether patients recognize that a decision is being made, understand the available options and their consequences, and have opportunities to express what matters to them and how they wish to participate in the decision [13].

A recent HSCT-specific framework illustrates how shared decision-making may be operationalized in practice. Olson et al. [53] proposed a three-step approach comprising individualized risk–benefit assessment, shared and supported decision-making, and ethical and practical implementation. Importantly, the framework extends beyond medical risk to include patients’ risk tolerance, values, relationships, social support, psychosocial and financial circumstances, and donor-related considerations. Although developed for preemptive HSCT in non-malignant hematological disorders and not yet validated as an intervention in hematological malignancies, it provides a useful conceptual example of how HSCT decision-making can integrate clinical evidence with the relational and practical contexts in which patients make decisions.

While the roles of the caregivers are vital across all oncological contexts, the present study indicates that in the context of hematological malignancies, the caregivers are not merely important but frequently an absolute prerequisite for initiating the transplantation [54–56]. Because transplantation centers mandate that the patients be accompanied by a 24-h caregiver for up to 100 days following the procedure [57], the medical decision inherently becomes a collective family mandate. Consequently, a clinical dilemma emerges: a medically viable, life-saving procedure often becomes practically unfeasible if it threatens to induce severe caregiver burnout or financial toxicity [55]. As our results and previous qualitative investigations [58] demonstrated, the psychological distress of becoming a familial burden can override the biological instinct to survive, compelling patients to prioritize collective family welfare over their individual cure. In light of this, it is imperative that comprehensive screening for social determinants of health be integrated into the transplant decision-making paradigm for both the patients and the caregivers. Systematically identifying potential barriers to family caregiving early on not only lessens these practical difficulties but also improves patients’ involvement in decision-making, guarantees subsequent therapeutic regimen compliance, and protects the health of the patient-caregiver pair [55].

Our findings also highlight that HSCT decision-making is not merely a clinical calculation, but a complex navigation of compounding socioeconomic and logistical barriers. Rather than viewing treatment costs and travel logistics as separate hurdles, this review underscores their deep intersection. The profound influence of prolonged travel, advanced coordination, and relocation observed in our findings aligns with the emerging concept of “time toxicity,” the substantial time burden patients incur while seeking care [59, 60]. Crucially, the synthesis demonstrates that for HSCT candidates, time toxicity inevitably translates into “financial toxicity” through out-of-pocket expenses and lost income [61]. This dual burden frequently acts as a definitive deterrent, forcing vulnerable populations to forgo potentially curative transplants in favor of localized, less intensive alternatives [62]. Given the significant influence of both financial and time toxicity on patients’ decisions, accurately identifying and measuring these factors is essential [63, 64]. Doing so would help healthcare professionals identify at-risk patients early, better understand their needs and preferences, and provide targeted support to mitigate the adverse effects of these dual burdens on decision-making [65].

Previous literature primarily emphasizes post-HSCT survivors’ pervasive anxieties regarding social reintegration—such as returning to employment or redefining social roles [66, 67]. In fact, the findings of the present review revealed that concerns about post-transplant social reintegration were already expressed by patients during the initial decision-making phase. Accordingly, eliciting and addressing patients’ concerns regarding fertility, occupational continuity, and bodily integrity during the pre-decision or decision-making phase may help alleviate their psychological burden and enable a more informed, value-concordant choice [68–70].

## Clinical implications

The findings of this review suggest that decision support for HSCT should first attend to whether patients genuinely perceive and understand the decision space, rather than focusing solely on the provision of treatment information or the completion of informed consent. For patients who perceive HSCT as the “only choice” or who tend to defer to clinicians, healthcare professionals should explore patients’ understanding of the available treatment options and the key trade-offs involved, as well as how they wish to participate in the decision-making process. For patients who prefer a clinician-led approach, this preference should likewise be clarified to ensure that it reflects an informed preference rather than passive acceptance.

The review also highlights that HSCT decisions may be constrained by practical circumstances, including caregiving availability, family responsibilities, financial burden, travel for treatment, and time commitments. Exploring these factors during the transplant evaluation process may help identify practical barriers beyond the medical considerations of transplantation and, where appropriate, facilitate access to relevant social, financial, or caregiving support.

In addition, patients were already considering the potential effects of HSCT on employment, fertility, physical functioning, social participation, and family roles when making their treatment decisions. Pre-transplant discussions may therefore benefit from addressing these longer-term concerns alongside survival, relapse, and transplant-related risks, allowing patients to consider how HSCT may affect the aspects of life they value most. Overall, these findings suggest that shared decision-making in HSCT should attend to patients’ understanding of available choices, the practical feasibility of transplantation, and their longer-term life goals, while the specific form and effectiveness of decision-support interventions remain to be established.

## Strengths and limitations

This review has several strengths. First, it focuses on patients’ subjective experiences during HSCT decision-making, rather than on post-transplant treatment experiences or survival outcomes alone, thereby providing a concentrated account of the concerns that matter to patients when accepting or declining transplantation. Second, by synthesizing qualitative studies across different hematological malignancy types, transplant modalities, and clinical contexts, it consolidates findings scattered across primary studies into four main themes: constrained choice, decision-making embedded in relational contexts, economic and logistical burdens, and concerns about social participation and identity change. This synthesis offers a more complete picture of the family, practical, and social considerations that shape HSCT decision-making beyond medical risks and benefits.

While this review offers a concentrated account of patients’ HSCT decision-making experiences, several limitations should nonetheless be acknowledged. First, the restriction to English-language publications may have excluded relevant evidence from other linguistic and cultural contexts. Although no lower limit was placed on publication year to capture the limited and fragmented evidence as comprehensively as possible, earlier studies may not fully reflect contemporary decision-making contexts given the continuous evolution of transplantation and supportive care; findings should therefore be interpreted in light of their year of publication. Second, most included studies asked patients to recall their decision-making experiences retrospectively, and some interviews were conducted 3 ~ 12 months post-transplant. Such accounts may be subject to recall bias and shaped by post-transplant experiences, limiting the representation of real-time decision-making. Future longitudinal qualitative studies could interview patients repeatedly across the decision-making trajectory. Finally, this review focused on the patient perspective and did not systematically integrate the experiences of family members, caregivers, or healthcare professionals, and thus primarily reflects how patients understand HSCT decision-making rather than the interactions among all parties involved. Future research could incorporate multiple perspectives to complement this understanding.

## Conclusion

This review shows that HSCT decision-making extends beyond clinical risks and benefits and is shaped by constrained choice, relational dependence, practical feasibility, and anticipated consequences for patients’ future lives. SDM-based decision support should therefore move beyond information provision to assess whether patients genuinely understand the decision space, identify practical circumstances that constrain choice, and address longer-term life concerns. Such support should also reflect patients’ preferred level of involvement and the relational contexts in which decisions are made. Future research should examine how patients’ perceptions and decision needs evolve across the HSCT decision-making trajectory and develop and evaluate decision-support interventions that better incorporate patients’ values, preferences, relational contexts, and practical circumstances.

## Supplementary Information

Below is the link to the electronic supplementary material.

Supplementary file1 (DOCX 13 kb)

Supplementary file2 (DOCX 12 kb)

Supplementary file3 (DOCX 17 kb)

## Data Availability

All data supporting the findings of this study are available within the paper and its Supplementary Information.

## References

[1] Zhang N, Wu J, Wang Q et al (2023) Global burden of hematologic malignancies and evolution patterns over the past 30 years. Blood Cancer J 13(1):82 10.1038/s41408-023-00853-3 PMC10188596 37193689

[2] An Z-Y, Fu H, He Y et al (2020) Projected global trends in hematological malignancies: incidence, mortality, and disability-adjusted life years from 2020 to 2030. Blood 142(Supplement 1):3810–3810

[3] Gray TF, Temel JS, El-Jawahri A (2021) Illness and prognostic understanding in patients with hematologic malignancies. Blood Rev 45:100692 10.1016/j.blre.2020.100692 32284227

[4] Mcerlean G, Ashley C, Pradhan A et al (2025) “It’s the ride of your life”: a qualitative study on survivor awareness and understanding of blood and marrow transplantation. Semin Oncol Nurs 41(1):151756 10.1016/j.soncn.2024.151756 39638666

[5] Spellman SR, Xu K, Oloyede T et al (2025) Current activity trends and outcomes in hematopoietic cell transplantation and cellular therapy - a report from the CIBMTR. Transplant Cell Ther 31(8):505–532 10.1016/j.jtct.2025.05.014 PMC12302970 40398621

[6] Randall J, Keven K, Atli T et al (2016) Process of allogeneic hematopoietic cell transplantation decision making for older adults. Bone Marrow Transplant 51(5):623–628 10.1038/bmt.2015.241 26457910

[7] Bhatt VR, Wang T, Chen K et al (2022) Chronic Graft-versus-Host Disease, nonrelapse mortality, and disease relapse in older versus younger adults undergoing matched allogeneic peripheral blood hematopoietic cell transplantation: a Center for International Blood and Marrow Transplant Research analysis. Transplant Cell Ther 28(1):34–42 10.1016/j.jtct.2021.10.002 PMC8792177 34637965

[8] Styczynski J, Tridello G, Koster L et al (2023) Decrease of lethal infectious complications in the context of causes of death (COD) after hematopoietic cell transplantation: COD-2 and COD-1 study of the Infectious Diseases Working Party EBMT. Bone Marrow Transplant 58(8):881–892 10.1038/s41409-023-01998-2 37149673

[9] Ruan J, Qian Y, Zhuang Y (2023) Survivorship experiences of Chinese hematopoietic stem cell transplantation survivors: a qualitative study [J]. Cancer Nurs. Accessed 13 Feb 2026 10.1097/NCC.0000000000001204 36727875

[10] Almutairi H, Aboshaiqah A, Alzahrani M et al (2025) The lived experience of adult cancer survivors after hematopoietic stem cell transplantation: a qualitative study from Saudi Arabia [J]. Patient Pref Adher 19(null):279–293 10.2147/PPA.S500554 PMC11794379 39911716

[11] Renzi C, Riva S, Masiero M et al (2016) The choice dilemma in chronic hematological conditions: why choosing is not only a medical issue? A psycho-cognitive perspective. Crit Rev Oncol Hematol 99:134–140 10.1016/j.critrevonc.2015.12.010 26762858

[12] Charles C, Gafni A, Whelan T (1997) Shared decision-making in the medical encounter: what does it mean? (or it takes at least two to tango) [J]. Soc Sci Med 44(5):681–692 10.1016/s0277-9536(96)00221-3 9032835

[13] Ottawa Hospital Research Institute. (2024) Patient decision aids research group [Z]. Accessed 20 Oct 2026

[14] Sepucha KR, Scholl I (2014) Measuring shared decision making. Circ Cardiovasc Qual Outcomes 7(4):620–626 10.1161/CIRCOUTCOMES.113.000350 24867916

[15] Pugh G, Young L, Yiallouridou C et al (2025) Understanding patient quality of life following haematopoietic cell transplant [J]. Support Care Cancer 33(7):562 10.1007/s00520-025-09578-4 PMC12152078 40493079

[16] Shi Y, Gao L, Yu JY et al (2023) Meta-synthesis of qualitative research on psychological experience and support needs of patients with hematopoietic stem cell transplantation in purified cabin at different treatment stages [J]. Med Inform 36(17):15–21, 33

[17] Bywater L, Atkins S (2001) A study of factors influencing patients’ decisions to undergo bone marrow transplantation from a sibling or matched related donor [J]. Eur J Oncol Nurs 5(1):7–15 10.1054/ejon.2000.0117 12849041

[18] Wu JF, Akinola IM, D’Souza A et al (2026) A multistakeholder qualitative analysis of barriers to autologous stem cell transplantation for multiple myeloma. Clin Lymphoma Myeloma Leuk 26(1):26–33 10.1016/j.clml.2025.07.002 PMC12357161 40744761

[19] Yuan X, Yang Y, Wang C et al (2024) Decision-making conflicts regarding hematopoietic stem cell transplantation in patients with hematological neoplasms: a descriptive qualitative study. Eur J Oncol Nurs. 10.1016/j.ejon.2024.102684 39163755

[20] Gurnari C, Robin M, Adès L et al (2025) Clinical-genomic profiling of MDS to inform allo-HCT: recommendations from an international panel on behalf of the EBMT. Blood 145(18):1987–2001 10.1182/blood.2024025131 39970324

[21] Cusatis RN, Tecca HR, D’Souza A et al (2020) Prevalence of decisional regret among patients who underwent allogeneic hematopoietic stem cell transplantation and associations with quality of life and clinical outcomes. Cancer 126(11):2679–2686 10.1002/cncr.32808 PMC7220834 32154926

[22] Liu SJ, Gu JX, Hu W et al (2021) Psychological experience of patients with hematopoietic stem cell transplantation: a meta-synthesis [J]. Chin Nurs Manag 21(07):1057–1063

[23] Crawford R, Sully K, Conroy R et al (2020) Patient-centered insights on treatment decision making and living with acute myeloid leukemia and other hematologic cancers. Patient 13(1):83–102 10.1007/s40271-019-00384-9 PMC6957575 31456136

[24] Nowak A, Nadolny S, Jahn F et al (2025) ‘I had no choice!’ Challenges in the informed consent and decision-making process for allogeneic stem cell transplantation: a qualitative method triangulation. Stem Cell Res Ther 16(1):623 10.1186/s13287-025-04766-9 PMC12593839 41199391

[25] Liu Q, Li J, Wang L et al (2025) Shared decision-making about autologous stem cell transplantation: a qualitative study of older patients and physicians [J]. J Am Geriatr Soc 73(2):458–469 10.1111/jgs.19276 39575972

[26] JBI (2020) The Joanna Briggs institute critical appraisal tools for use in JBI systematic reviews: checklist for qualitative research[Z]. Accessed 20 Mar 2026

[27] Tong A, Flemming K, Mcinnes E et al (2012) Enhancing transparency in reporting the synthesis of qualitative research: ENTREQ. BMC Med Res Methodol 12(1):181 10.1186/1471-2288-12-181 PMC3552766 23185978

[28] Sandelowski M (2007) Handbook for synthesizing qualitative research. springer publishing company

[29] Elo S, Kyngäs H (2008) The qualitative content analysis process. J Adv Nurs 62(1):107–115 10.1111/j.1365-2648.2007.04569.x 18352969

[30] Lauri S, Kyngäs H, Honkala S et al (2005) Hoitotieteen teorian kehittäminen, 1. p. ed. WSOY, Porvoo ; Helsinki

[31] Lewin S, Glenton C, Munthe-Kaas H et al (2015) Using qualitative evidence in decision making for health and social interventions: an approach to assess confidence in findings from qualitative evidence syntheses (GRADE-CERQual). PLoS Med 12(10):e1001895 10.1371/journal.pmed.1001895 PMC4624425 26506244

[32] Fiala MA, Vij R, Wildes TM (2019) A mixed-methods study of stem cell transplantation utilization for newly diagnosed multiple myeloma. Clin Lymphoma Myeloma Leuk 19(9):e521–e525 10.1016/j.clml.2019.05.003 31320252

[33] Forsyth R, Scanlan C, Carter SM et al (2011) Decision making in a crowded room: the relational significance of social roles in decisions to proceed with allogeneic stem cell transplantation [J]. Qual Health Res 21(9):1260–1272 10.1177/1049732311405802 21471426

[34] Hansen DK, Bixby T, Fixler K et al (2025) Patient and caregiver perspectives on treatment decision-making for stem cell transplantation in multiple myeloma. Support Care Cancer 33(2):134 10.1007/s00520-025-09195-1 39890679

[35] Nguyen TQ, Le TD, Fan S-Y et al (2025) Exploring relational autonomy of Vietnamese patients’ experiences in decision-making regarding hematopoietic stem cell transplantation: a qualitative interview study. Psychooncology 34(6):e70185 10.1002/pon.70185 40437648

[36] Sarradon-Eck A, Franchina L, Arnault Y et al (2024) Consenting rather than choosing. A qualitative study on overseas patients’ decision to undergo hematopoietic stem cell transplantation [J]. Cancer Med 13(1):e6934 10.1002/cam4.6934 PMC10807688 38193147

[37] Yanez B, Taub CJ, Waltz M et al (2023) Stem cell transplant experiences among Hispanic/Latinx patients: a qualitative analysis. Int J Behav Med 30(5):628–638 10.1007/s12529-022-10126-1 PMC9589807 36266388

[38] Reimer A, Schepers C, Ley M et al (2025) Navigating what is not known—patients’ experience of the life threat connected to allogeneic stem cell transplantation. Eur J Cancer Care 2025(1):8743444

[39] Foster J, Moore H, Preussler JM et al (2020) Information needs for treatment decision-making of hematopoietic cell transplant patients 65 years or older and caregivers. J Cancer Educ 35(4):651–660 10.1007/s13187-019-01506-5 30877651

[40] Solanki H, Tiwari AK, Vashisht N et al (2021) Barriers and support-system while considering hematopoietic stem cell transplant (HSCT): a qualitative study of pre-HSCT acute leukemia patients from a standalone transplant laboratory in India. Indian J Med Paediatr Oncol 42(02):153–160

[41] Mian O, Puts M, Mccurdy A et al (2022) Decision-making factors for an autologous stem cell transplant for older adults with newly diagnosed multiple myeloma: a qualitative analysis [J]. Front Oncol 12. Accessed 13 Feb 2026 10.3389/fonc.2022.974038 PMC9911655 36776355

[42] Cytryn R, Bickell N, Yagnik R et al (2023) What affects treatment underuse in multiple myeloma in the United States: a qualitative study. Cancers (Basel) 15(8):2369 10.3390/cancers15082369 PMC10137271 37190297

[43] Hansen DK, Bixby T, Fixler K et al (2023) Experience of autologous stem cell transplant in multiple myeloma: the patient and caregiver perspective [J]. J Patient Exp 10:23743735231202732 10.1177/23743735231202733 PMC10521273 37766811

[44] Fadem S (2021) Design for sensemaking in complex and ambiguous medical situations [D]. United States -- New Jersey; Rutgers The State University of New Jersey, School of Graduate Studies. Accessed 13 Feb 2026

[45] Kumasaka LMKB (1991) Factors influencing hazardous decision making related to bone marrow transplant [D]. United States -- Hawaii; University of Hawai'i at Manoa. Accessed 13 Feb 2026

[46] Haberman MR (1987) Living with leukemia: the personal meaning attributed to illness and treatment by adults undergoing a bone marrow transplantation [D]. United States -- Washington; University of Washington. Accessed 13 Feb 2026

[47] Spierings J, VAN Rhijn-Brouwer FCC, DE Bresser CJM et al (2020) Treatment decision-making in diffuse cutaneous systemic sclerosis: a patient’s perspective. Rheumatology (Oxford) 59(8):2052–2061 10.1093/rheumatology/kez579 PMC7382600 31808528

[48] Bakshi N, Katoch D, Sinha CB et al (2020) Assessment of patient and caregiver attitudes and approaches to decision-making regarding bone marrow transplant for sickle cell disease: a qualitative study [J]. JAMA Netw Open 3(5):e206742–e206742 10.1001/jamanetworkopen.2020.6742 PMC7260617 32469414

[49] Leuthold N, Cattaneo M, Halter J et al (2021) Patient preferences for allogeneic haematopoietic stem cell transplantation: how much benefit is worthwhile from the patient’s perspective? Support Care Cancer 29(6):3129–3135 10.1007/s00520-020-05816-z PMC8062338 33067766

[50] Yu X, Xiao Z, Li X et al (2025) From risk perception to action: a qualitative study of self-management in hematopoietic stem cell transplant patients. Cell Transplant 34:9636897251393308 10.1177/09636897251393308 PMC12681589 41342251

[51] Ernst J, Holze S, Sonnefeld C et al (2007) Medical decision making in hospital - results of an exploratory study on the value of shared decision making from the physicians point of view [J]. Gesundheitswesen 69(4):206–215 10.1055/s-2007-973091 17533562

[52] Patenaude AF, Rappeport JM, Smith BR (1986) The physician’s influence on informed consent for bone marrow transplantation. Theor Med Bioeth 7(2):165–179 10.1007/BF00489228 3526626

[53] Olson TS, Ericson K, Antin JH et al (2026) Preemptive hematopoietic stem cell transplantation in RUNX1 familial platelet disorder: a shared decision-making framework [J]. Haematologica 111(9):2911–2923 10.3324/haematol.2025.300403 PMC13530962 41924923

[54] Preussler JM, Mau L-W, Majhail NS et al (2019) Caregiver availability and patient access to hematopoietic cell transplantation: social worker perspectives inform practice. Support Care Cancer 27(11):4253–4264 10.1007/s00520-019-04696-2 30850889

[55] Tweeten B, Randall J, Barata A et al (2025) The caregiver paradigm in hematopoietic cell transplant: current and future directions. Transplant Cell Ther 31(11):874–888 10.1016/j.jtct.2025.06.022 PMC12395758 40544999

[56] Anna Desalvo M, CGC, Jaime Preussler, MS (2023) The caregiver impact on access to alloHCT treatment [Z]. Cellgene. Accessed 14 Feb 2026

[57] Preussler JM, Desalvo AM, Huberty P et al (2024) Can you spare 100 days? The allogeneic hematopoietic cell transplant caregiver requirement from the perspective of social workers. Support Care Cancer 32(11):723 10.1007/s00520-024-08906-4 PMC12532042 39395065

[58] Pritlove C, Jassi M, Burns B et al (2021) The work of managing multiple myeloma and its implications for treatment-related decision making: a qualitative study of patient and caregiver experiences. BMC Cancer 21(1):793 10.1186/s12885-021-08527-8 PMC8268411 34238260

[59] Gupta A, Brundage MD, Galica J et al (2024) Patients’ considerations of time toxicity when assessing cancer treatments with marginal benefit. Oncologist 29(11):978–985 10.1093/oncolo/oyae187 PMC11546709 39045654

[60] Nwozichi C, Omolabake S, Ojewale MO et al (2024) Time toxicity in cancer care: a concept analysis using Walker and Avant’s method. Asia Pac J Oncol Nurs. 10.1016/j.apjon.2024.100610 PMC11617379 39641009

[61] Iragorri N, DE Oliveira C, Fitzgerald N et al (2021) The out-of-pocket cost burden of cancer care-a systematic literature review. Curr Oncol 28(2):1216–1248 10.3390/curroncol28020117 PMC8025828 33804288

[62] Parker C, Berkovic D, Ayton D et al (2022) Patient perceived financial burden in haematological malignancies: a systematic review. Curr Oncol 29(6):3807–3824 10.3390/curroncol29060305 PMC9221876 35735414

[63] Greenup RA (2022) Financial toxicity and shared decision making in oncology. Surg Oncol Clin N Am 31(1):1–7 10.1016/j.soc.2021.08.001 34776059

[64] Fiala MA (2024) Financial toxicity and willingness-to-pay for cancer treatment among people with multiple myeloma. JCO Oncol Pract 20(9):1263–1271 10.1200/OP.24.00016 38885465

[65] Jing J, Li M, Rui Y et al (2025) The weight of time: experience of time toxicity among advanced cancer patients and their family caregivers-a qualitative study. Support Care Cancer 33(10):862 10.1007/s00520-025-09904-w 40968315

[66] Liu X, Hu W, Wei Y (2024) Research progress on returning to work among survivors of hematopoietic stem cell transplantation [J]. Chin Nurs Manag 24(10):1586–1591

[67] Majhail NS (2017) Long-term complications after hematopoietic cell transplantation. Hematol Oncol Stem Cell Ther 10(4):220–227 10.1016/j.hemonc.2017.05.009 PMC5925745 28641097

[68] DE Boer AG, Taskila TK, Tamminga SJ et al (2015) Interventions to enhance return-to-work for cancer patients. Cochrane Database Syst Rev. 10.1002/14651858.cd007569.pub3 PMC6483290 26405010

[69] Salit RB, Schoeppner K, DE Biase C et al (2022) American Society for Transplantation and Cellular Therapy Return to Work Guidance Committee recommendations for health care providers who take care of hematopoietic cell transplantation patients. Transplant Cell Ther 28(12):822–828 10.1016/j.jtct.2022.09.017 36184059

[70] Kurosawa S, Yamaguchi T, Mori A et al (2022) Resignation and return to work in patients receiving allogeneic hematopoietic cell transplantation close up [J]. J Cancer Surviv 16(5):1004–1015 10.1007/s11764-021-01092-w 34449050

