# Supplementary material for: The perceptions of hematopoietic stem cell transplantation decision-making in patients with hematologic malignancies: a qualitative systematic review
Source: Support Care Cancer. 2026 Oct 23;34(11):1149. doi: 10.1007/s00520-026-11381-8 (PMC13694318; doi:10.1007/s00520-026-11381-8)
Supplement: Supplementary file 1 [file 520_2026_11381_MOESM1_ESM.docx]

**Supplement 1 Searching Strategies**

| Database | Literature | Search Strategy |
| --- | --- | --- |
| PubMed | 387 | ((((((("decision"[Title/Abstract]) OR ("decision mak*"[Title/Abstract])) OR (choice[Title/Abstract])) OR ("decision-making"[Title/Abstract])) ()) AND ("Qualitative Research" [MeSH Terms] OR "Grounded Theory" [MeSH Terms] OR "qualitative study" [Title/Abstract] OR "qualitative" [Title/Abstract] OR "phenomenon" [Title/Abstract] OR "mixed methods" [Title/Abstract] OR "content analysis" [Title/Abstract] OR "descriptive study" [Title/Abstract] OR "view" [Title/Abstract] OR "cognition" [Title/Abstract] OR "experience" [Title/Abstract] OR "feeling" [Title/Abstract] OR "psychology" [Title/Abstract] OR "perspectives" [Title/Abstract] OR "focus group" [Title/Abstract] OR "interview" [Title/Abstract] OR "attitude" [Title/Abstract])) ()) AND ((("Hematopoietic stem cell transplantation"[Title/Abstract]) OR ("stem cell transplantation"[Title/Abstract])) OR ("bone marrow transplantation"[Title/Abstract])) |
| Web Of Science | 594 | TS=(("decision" OR "decision mak*" OR "choice" OR "decision-making") AND ("Hematopoietic stem cell transplantation" OR "stem cell transplantation" OR "bone marrow transplantation" OR "stem cell based therapy" OR "stem cell therapy" OR "transplantation stem cell" OR "bone marrow cell transfer" OR "bone marrow graft" OR "bone marrow grafting" OR "bone marrow transfusion" OR "bone marrow transplant" OR "transplantation bone marrow") AND ("Qualitative Research" OR "Grounded Theory" OR "qualitative study" OR "qualitative" OR "phenomenon" OR "mixed methods" OR "content analysis" OR "descriptive study" OR "view" OR "cognition" OR "experience" OR "feeling" OR "psychology" OR "perspectives" OR "focus group" OR "interview" OR "attitude")) |
| Embase | 682 | (('qualitative research':ab,ti OR 'grounded theory':ab,ti OR 'qualitative study':ab,ti OR 'qualitative':ab,ti OR 'phenomenon':ab,ti OR 'mixed methods':ab,ti OR 'content analysis':ab,ti OR 'descriptive study':ab,ti OR 'view':ab,ti OR 'cognition':ab,ti OR 'experience':ab,ti OR 'feeling':ab,ti OR 'psychology':ab,ti OR 'perspectives':ab,ti OR 'focus group':ab,ti OR 'interview':ab,ti OR 'attitude':ab,ti) AND [embase]/lim) AND ((('stem cell transplantation'/exp OR 'stem cell transplantation') AND [embase]/lim) OR (('bone marrow transplantation'/exp OR 'bone marrow transplantation') AND [embase]/lim) OR (('stem cell based therapy':ab,ti OR 'stem cell therapy':ab,ti OR 'transplantation, stem cell':ab,ti OR 'stem cell transplantation':ab,ti OR 'bone marrow cell transfer':ab,ti OR 'bone marrow graft':ab,ti OR 'bone marrow grafting':ab,ti OR 'bone marrow transfusion':ab,ti OR 'bone marrow transplant':ab,ti OR 'transplantation, bone marrow':ab,ti OR 'bone marrow transplantation':ab,ti) AND [embase]/lim)) AND ((('decision making'/exp OR 'decision making') AND [embase]/lim) OR (('arbitration':ab,ti OR 'career choice':ab,ti OR 'choice behavior':ab,ti OR 'choice behaviour':ab,ti OR 'choice making':ab,ti OR 'decision process':ab,ti OR 'decisionmaking':ab,ti OR 'judgement':ab,ti OR 'judgment':ab,ti OR 'decision making':ab,ti) AND [embase]/lim)) |
| CINAHL | 89 | XB ("Hematopoietic stem cell transplantation" OR "stem cell transplantation" OR "bone marrow transplantation" OR "stem cell based therapy" OR "stem cell therapy" OR "transplantation stem cell" OR "bone marrow cell transfer" OR "bone marrow graft" OR "bone marrow grafting" OR "bone marrow transfusion" OR "bone marrow transplant" OR "transplantation bone marrow") AND XB ("decision" OR "decision mak*" OR "choice" OR "decision-making") AND XB ("Qualitative Research" OR "Grounded Theory" OR "qualitative study" OR "qualitative" OR "phenomenon" OR "mixed methods" OR "content analysis" OR "descriptive study" OR "view" OR "cognition" OR "experience" OR "feeling" OR "psychology" OR "perspectives" OR "focus group" OR "interview" OR "attitude") |
| Scopus | 1,908 | TITLE-ABS-KEY ( "Hematopoietic stem cell transplantation" OR "stem cell transplantation" OR "bone marrow transplantation" OR "stem cell based therapy" OR "stem cell therapy" OR "transplantation stem cell" OR "bone marrow cell transfer" OR "bone marrow graft" OR "bone marrow grafting" OR "bone marrow transfusion" OR "bone marrow transplant" OR "transplantation bone marrow" ) AND TITLE-ABS-KEY ( "Qualitative Research" OR "Grounded Theory" OR "qualitative study" OR "qualitative" OR "phenomenon" OR "mixed methods" OR "content analysis" OR "descriptive study" OR "view" OR "cognition" OR "experience" OR "feeling" OR "psychology" OR "perspectives" OR "focus group" OR "interview" OR "attitude" ) AND TITLE-ABS-KEY ( "decision" OR "decision mak*" OR "choice" OR "decision-making" ) |
| ProQuest Dissertations & Theses Global | 35 | summary("decision" OR "decision mak*" OR "choice" OR "decision-making" OR “choos*”) AND summary("Qualitative Research" OR "Grounded Theory" OR "qualitative study" OR "qualitative" OR "phenomenon" OR "mixed methods" OR "content analysis" OR "descriptive study" OR "view*" OR "cognition" OR "experienc*" OR "feeling" OR "psychology" OR "perspectiv*" OR "focus group" OR "interview" OR "attitud*" OR perception* OR opinion) AND summary("Hematopoietic stem cell transplantation" OR "stem cell transplantation" OR "bone marrow transplantation" OR "stem cell based therapy" OR "stem cell therapy" OR "transplantation stem cell" OR "bone marrow cell transfer" OR "bone marrow graft" OR "bone marrow grafting" OR "bone marrow transfusion" OR "bone marrow transplant" OR "transplantation bone marrow") |
| Cochrane Library | 111 | #1 MeSH descriptor: [Hematopoietic Stem Cell Transplantation] explode all trees #2 ('stem cell based therapy' OR 'stem cell therapy' OR 'transplantation, stem cell' OR 'stem cell transplantation' OR 'bone marrow cell transfer' OR 'bone marrow graft' OR 'bone marrow grafting' OR 'bone marrow transfusion' OR 'bone marrow transplant' OR 'transplantation, bone marrow' OR 'bone marrow transplantation'):ti,ab,kw #3 #1 OR #2 #4 MeSH descriptor: [Decision Making] explode all trees #5 (decision OR decision NEXT making OR choice):ti,ab,kw #6 MeSH descriptor: [Qualitative Research] explode all trees #7 ("Qualitative Research" OR "Grounded Theory" OR "qualitative study" OR "qualitative" OR "phenomenon" OR "mixed methods" OR "content analysis" OR "descriptive study" OR "view" OR "cognition" OR "experience" OR "feeling" OR "psychology" OR "perspectives" OR "focus group" OR "interview" OR "attitude"):ti,ab,kw #8 #7 OR #7 #9 #4 OR #5 #10 #3 AND #8 AND #9 |
| PsycARTICLES | 5 | XB ("Hematopoietic stem cell transplantation" OR "stem cell transplantation" OR "bone marrow transplantation" OR "stem cell based therapy" OR "stem cell therapy" OR "transplantation stem cell" OR "bone marrow cell transfer" OR "bone marrow graft" OR "bone marrow grafting" OR "bone marrow transfusion" OR "bone marrow transplant" OR "transplantation bone marrow") AND XB ("decision" OR "decision mak*" OR "choice" OR "decision-making") AND XB ("Qualitative Research" OR "Grounded Theory" OR "qualitative study" OR "qualitative" OR "phenomenon" OR "mixed methods" OR "content analysis" OR "descriptive study" OR "view" OR "cognition" OR "experience" OR "feeling" OR "psychology" OR "perspectives" OR "focus group" OR "interview" OR "attitude") |
