# Supplementary material for: The perceptions of hematopoietic stem cell transplantation decision-making in patients with hematologic malignancies: a qualitative systematic review
Source: Support Care Cancer. 2026 Oct 23;34(11):1149. doi: 10.1007/s00520-026-11381-8 (PMC13694318; doi:10.1007/s00520-026-11381-8)
Supplement: Supplementary file 2 [file 520_2026_11381_MOESM2_ESM.docx]

**Supplementary File 2 Confidence in Evidence for Qualitative Research Methodology Reviews**

| **Category** | **Research Contribution** | **CERQual Confidence Level** | **CERQual Evaluation Explanation** |
| --- | --- | --- | --- |
| **Constrained choices** | Bywater & Atkins, 2001; Fadem, 2021; Forsyth et al., 2011; Haberman, 1987; Kumasaka, 1991; Mian et al., 2023; Nowak et al., 2025; Reimer et al., 2025; Sarradon-Eck et al., 2024; Solanki et al., 2021; Yuan et al., 2024 | High Confidence | Minor concerns regarding methodological limitations, coherence, relevance, and adequacy of data. |
| **Decision-making embedded in relational contexts** | Forsyth et al., 2011; Foster et al., 2020; Solanki et al., 2021; Hansen et al., 2023; Mian et al., 2023; Yanez et al., 2023; Liu et al., 2025; Nguyen et al., 2025; Sarradon-Eck et al., 2024; Wu et al., 2026; Yuan et al., 2024 | High Confidence | Minor concerns regarding methodological limitations, coherence, relevance, and adequacy of data. |
| **Concerns about restricted social participation and threats to identity caused by transplant** | Foster et al., 2020; Solanki et al., 2021; Yuan et al., 2024; Fiala et al., 2019; Sarradon-Eck et al., 2024 | Moderate Confidence | Moderate concerns regarding methodological limitations and adequacy of data. Minor concerns regarding coherence and relevance. |
| **The hidden toll: Financial constraints and logistical hurdles** | Cytryn et al., 2023; Liu et al., 2025; Sarradon-Eck et al., 2024; Solanki et al., 2021; Wu et al., 2026; Yanez et al., 2023 | Low Confidence | Moderate  concerns regarding relevance and  substantial concerns regarding  adequacy of data. |
