# Supplementary material for: The perceptions of hematopoietic stem cell transplantation decision-making in patients with hematologic malignancies: a qualitative systematic review
Source: Support Care Cancer. 2026 Oct 23;34(11):1149. doi: 10.1007/s00520-026-11381-8 (PMC13694318; doi:10.1007/s00520-026-11381-8)
Supplement: Supplementary file 3 [file 520_2026_11381_MOESM3_ESM.docx]

Table 2：Results of the critical appraisal of the included studies

| Author，Year | Q1 | Q2 | Q3 | Q4 | Q5 | Q6 | Q7 | Q8 | Q9 | Q10 |
| --- | --- | --- | --- | --- | --- | --- | --- | --- | --- | --- |
| Bywater and Atkins (2001) | Y | Y | Y | Y | Y | Y | N | Y | Y | Y |
| Cytryn et al. (2023) | U | Y | Y | Y | Y | Y | N | Y | Y | Y |
| Fadem et al (2021) | N | Y | Y | Y | Y | U | N | Y | Y | Y |
| Fiala et al. (2019) | Y | Y | Y | Y | Y | Y | Y | Y | Y | Y |
| Forsyth et al. (2011) | Y | Y | Y | Y | Y | Y | N | Y | Y | Y |
| Foster et al. (2020) | U | Y | Y | Y | Y | N | N | Y | Y | Y |
| Haberman (1987) | N | Y | Y | Y | Y | U | N | Y | Y | Y |
| Hansen et al. (2023) | U | Y | Y | Y | Y | Y | N | Y | Y | Y |
| Hansen et al. (2025) | Y | Y | Y | Y | Y | N | N | Y | Y | Y |
| Kumasaka (1991) | N | Y | Y | Y | Y | U | N | Y | Y | Y |
| Liu et al. (2025) | U | Y | Y | Y | Y | Y | N | Y | Y | Y |
| Mian et al. (2023) | U | Y | Y | Y | Y | Y | N | Y | Y | Y |
| Nguyen et al. (2025) | Y | Y | Y | Y | Y | Y | U | Y | Y | Y |
| Nowak et al. (2025) | U | Y | Y | Y | Y | Y | U | Y | Y | Y |
| Reimer et al. (2025) | Y | Y | Y | Y | Y | Y | Y | Y | Y | Y |
| Sarradon-Eck et al. (2024) | Y | Y | Y | Y | Y | Y | Y | Y | Y | Y |
| Solanki et al. (2021) | U | Y | Y | Y | Y | Y | N | Y | Y | Y |
| Wu et al. (2026) | U | Y | Y | Y | Y | Y | N | Y | Y | Y |
| Yanez et al. (2023) | Y | Y | Y | Y | Y | Y | N | Y | Y | Y |
| Yuan et al. (2024) | Y | Y | Y | Y | Y | Y | Y | Y | Y | Y |

*Note*: Y, yes; N, no; U, unclear.

Q1 Is there congruity between the stated philosophical perspective and the research methodology?

Q2 Is there congruity between the research methodology and the research question or objectives?

Q3 Is there congruity between the research methodology and the methods used to collect data?

Q4 Is there congruity between the research methodology and the representation and analysis of data?

Q5 Is there congruity between the research methodology and the interpretation of results?

Q6 Is there a statement locating the researcher culturally or theoretically?

Q7 Is the influence of the researcher on the research, and vice versa, addressed?

Q8 Are participants, and their voices, adequately represented?

Q9 Is the research ethical according to current criteria or, for recent studies, and is there evidence of ethical approval by an appropriate body?

Q10 Do the conclusions drawn in the research report flow from the analysis, or interpretation, of the data?
